# Calcified gallstone in a 3 year-old boy: a case report

**DOI:** 10.1186/1756-0500-5-433

**Published:** 2012-08-13

**Authors:** Erik R Barthel, James R Pierce, Osnat Zmora, Susan R Harlan, Sudha Russell, Cathy Shin

**Affiliations:** 1 Division of Pediatric Surgery, Children’s Hospital Los Angeles, 4650 Sunset Blvd Mailstop 100, Los Angeles, CA 90027, USA; 2 Department of Radiology, Children’s Hospital Los Angeles, 4650 Sunset Blvd Mailstop 81, Los Angeles, CA, 90027, USA; 3 Department of Emergency and Transport Medicine, Children's Hospital Los Angeles, 4650 Sunset Blvd Mailstop 113, Los Angeles, CA, 90027, USA

## Abstract

**Background:**

Gallstones are relatively rare in children. At-risk populations include patients suffering from hemolysis syndromes. Regardless of etiology, these patients usually will present with postprandial abdominal pain, and ultrasonography is the mainstay of diagnosis. However, some gallstones are radiopaque and can be visualized on plain abdominal radiography.

**Case presentation:**

We present the uncommon but classic plain x-ray finding of a calcified gallstone in a 3 year-old Hispanic boy. He was treated with elective laparoscopic cholecystectomy.

**Conclusions:**

Cholelithiasis is rare in children, and calcified stones that will appear on plain abdominal x-rays are even rarer. If symptomatic, cholecystectomy by a pediatric surgeon is the treatment of choice. We discuss some of the recent developments in treatment of this condition in this patient population.

## Background

Although not common in children, gallstones are seen with increased incidence in pediatric patients with hematologic disorders [1]. In adults, an increased risk of gallstones is seen with obesity [2]. Given the rising incidence of obesity in children in the United States, it is likely that cholelithiasis will also increase in this population with time [3]. There are relatively sparse reports in the literature of a calcified gallstone in the pediatric population [4].

## Case presentation

**Figure 1:**
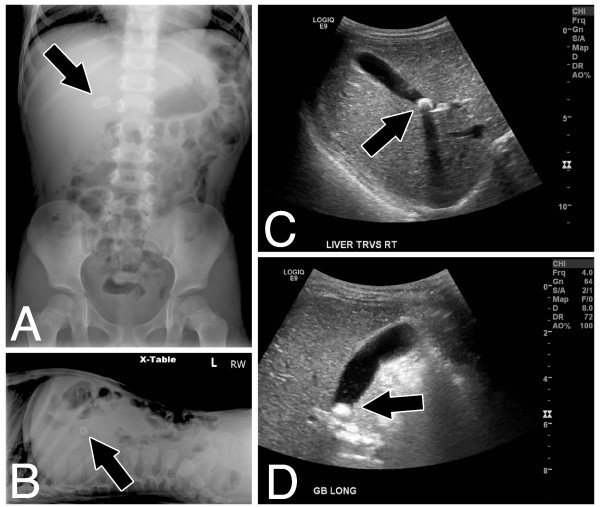
**Panel A: anterior/posterior abdominal radiograph.** Panel **B**: Cross-table abdominal radiograph. Panels **C**, **D**: Transverse and longitudinal ultrasound views of liver and gallbladder. Arrows indicate the gallstone.

An otherwise healthy 3 years and 10 months-old, 99 cm, 14 kg Hispanic boy presented to our Emergency Department after multiple visits to other facilities during the same week with a chief complaint of abdominal pain. The pain was described as postprandial and crampy, and the child’s mother endorsed his aversion to fatty foods and carbonated beverages. She denied emesis, jaundice, icterus, pruritus, or symptoms of altered mental status. Physical examination was unremarkable. Laboratory studies showed only borderline elevated total bilirubin. A plain abdominal x-ray was obtained, which revealed a radiodense mass in the right upper quadrant (Figure 1, panels A, B, arrows). The patient was diagnosed with calcified gallstone. This was confirmed with an abdominal ultrasound, which further showed that the 1.2 × 0.8 × 0.7 cm stone was impacted in the neck of the gallbladder (Figure 1, panels C, D, arrows). Elective cholecystectomy was performed using the standard four-port laparoscopic technique, with the finding of a large pigmented stone in the gallbladder neck.

## Discussion

Fifteen percent of gallstones are sufficiently calcified to be radiodense enough to be visualized on plain radiographs, and of these, two thirds are pigment stones [5]. In general, gallstones are uncommon in children, with patients under 15 comprising only 0.1-0.2% of the incidence of the disease [6]. In the pediatric population, pigment stones containing bilirubin salts are more common [7]. These types of stones are associated with hemolytic disorders, most commonly sickle cell anemia [8]. In our patient, after his cholecystectomy a hematologic workup was recommended to his primary physician. Laparoscopic cholecystectomy can be safely performed in the case of symptomatic cholelithiasis in this population. It is the treatment of choice for gallstones in children with sickle cell disease [9]. Moreover, it can be safely performed as an outpatient procedure, rather than having to incur the additional costs of an overnight hospital stay [10]. More recently, the use of a single-incision approach, in place of the traditional 4-trocar laparoscopic cholecystectomy employed here, has been applied to pediatric patients. These authors note that the single-incision method can be used in children for a variety of laparoscopic procedures, and though it carries some drawbacks, many of these can be overcome by employing specially adapted instruments [11].

## Conclusion

Despite its relative rarity in comparison to adults, cholelithiasis must always be on the differential diagnosis with a childhood complaint of postprandial abdominal pain. Cholecystectomy is the appropriate treatment for symptomatic cholelithiasis, especially so in children with sickle cell disease or other hemolytic disorders. It can be performed safely in the outpatient setting, and the emerging technique of single-incision laparoscopy will likely play an increasingly important role in its management in carefully selected patients.

## Consent

Written informed consent was obtained from this minor patient’s parents for publication of this case report and the accompanying images. A de-identified copy of the written consent is available for review by the Editor-in-Chief of this journal.

## Competing interests

The authors declare that they have no competing interests.

## Authors’ contributions

ERB, JRP and CS performed the literature search and wrote the manuscript. CS and OZ performed the surgeries, and SR and SRH provided radiologic images. All authors have read, critically reviewed, and approved the final manuscript.
